# Yttrium Copper Titanate as a Highly Efficient Electrocatalyst for Oxygen Reduction Reaction in Fuel Cells, Synthesized via Ultrafast Automatic Flame Technique

**DOI:** 10.1038/s41598-017-09661-9

**Published:** 2017-08-24

**Authors:** Laxman Singh, Uday Pratap Azad, Satendra Pal Singh, Vellaichamy Ganesan, U. S. Rai, Youngil Lee

**Affiliations:** 1 Department of Chemistry, University of Ulsan, 93 Daehak-ro Nam-gu, Ulsan, 44610 Republic of Korea; 2 Department of Chemistry, Institute of Science, Banaras Hindu University, Varanasi, 221005 Uttar Pradesh India; 3 Faculty of Nanotechnology and Advanced Materials Engineering, Sejong University, Seoul, 05006 Republic of Korea

## Abstract

Replacing platinum (Pt) metal-based electrocatalysts used in the oxygen reduction reaction (ORR) in fuel cells is an important research topic due to the high cost and scarcity of Pt, which have restricted the commercialization of these clean-energy technologies. The ABO_3_-type perovskite family of an ACu_3_Ti_4_O_12_ (A = Ca, Y, Bi, and La) polycrystalline material can serve as an alternative electrocatalyst for the ORR in terms of low-cost, activity, and stability. These perovskite materials may be considered the next generation electro-catalyst for the ORR because of their photocatalytic activity and physical and chemical properties capable of containing a wide range of A- and B-site metals. This paper reports the ORR activity of a new Y_2/3_Cu_3_Ti_4_O_12_ perovskite, synthesized via a rapid and facile automatic flame synthesis technique using rotating disk electrode (RDE) measurements. Y_2/3_Cu_3_Ti_4_O_12_/C has superior ORR activity, stability, and durability compared to commercial Pt/C. The results presented in this article will provide the future perspectives to research based on ACu_3_Ti_4_O_12_ (A = Ca, Y, Bi, Sm, Cd, and La) perovskite as the next generation electro-catalyst for the ORR in various electrochemical devices, such as fuel cells, metal–air batteries, and electrolysis.

## Introduction

Fuel cells (FCs) are a new power source via the direct conversion of hydrogen to electricity as a potential replacement for Li-ion batteries systems in terms of safety, high efficiency, renewable sources, and environmental friendliness^1^. The major obstacle to the commercialization of fuel cells is the high cost, poor stability, and slow kinetics of the oxygen reduction reaction (ORR) of platinum and platinum-based electrocatalysts in fuel-cell electrodes^2^. Thus far, the best electrocatalysts for the ORR of the cathode are carbon-supported Pt and/or its composites^3–8^. High cost and scarcity of platinum requires either use of noble metal with an increased efficiency or the utilization of non-precious electrocatalysts for commercialization on a large scale. In addition, Pt-based electrocatalysts suffer from methanol crossover and CO poisoning^9^. The ORR is not only an essential electrochemical process in fuel cells, but is also required for other electrochemical technologies, such as metal-air batteries and water electrolysis^10^. The ORR takes place through multiple electron transfer in alkaline media. Depending on the nature and electrocatalytic activity of the catalysts, the ORR in alkaline media can occur either via a two electron process to produce HO_2_^−^ or a four electron process to produce OH^−^ (O_2_ + 2H_2_O + 4e^−^ → 4OH^−^)^11^. A high ORR overpotential has been the main obstacle to making these technologies viable. Therefore, major efforts have been made to discover cost-effective and efficient ORR catalysts in traditional aqueous media. A general model of the oxygen reduction kinetics in porous electrodes must include oxygen diffusion, oxygen adsorption, or surface reaction on the active sites of the catalyst, charge transfer, and the diffusion of products^12^. ABO_3_ type perovskite oxides, particularly ACu_3_Ti_4_O_12_ (A = Ca, La, Bi, Sm, Cd, and Y), have great potential as low cost, high stability, and better kinetics electro-catalyst and may be considered the next generation electro-catalyst for the ORR because of their photocatalytic activity and physical and chemical properties capable of containing a wide variety of A- and B-site metals. These oxide materials have been studied extensively towards a wide range of applications, such as microelectronics, ceramic capacitors, dynamic random-access memory, transducers, microwave device applications, and other electronic devices^13, 14^. Currently, the ORR activity of perovskite electrocatalysts has attracted considerable attention^15–18^. The ORR activity of various perovskite materials proceeds through four electron transfer. Different hypothesis for the ORR activity of the perovskite materials have been proposed. Bockris *et al*.^19^ suggested that the ORR activity is accompanied by the presence of transition metal d-electrons and the strength of the M-OH bond during the rate determining steps. Suntivich *et al*.^20^ reported that the “activity descriptor” governing the ORR activity in transition metal oxides is determined by the extent of σ*-anti bonding (e_g_) orbital filling of the metal ions on the surface. Matsumoto *et al*.^21^ proposed the formation of a σ* bond. The high ORR activity is related to the higher oxidation state of transition metal cations. Until now, the proposed mechanisms are still persisting, which govern the ORR activity in perovskite materials. Various forms of graphene and its nano-composite with metal oxide/metal nanoparticles have been used extensively for FCs. Very few studies have examined the direct use of perovskite and ceramic-based materials for the ORR in FC. Yagi *et al*.^22^ showed that the covalent bonding network in the A (Cu^2+^) and B site (Fe^4+^) metal cations improves the structural stability of CaCu_3_Fe_4_O_12_ increasing the highly active long-life catalysts for the oxygen evolution reaction. Chen *et al*.^23^ reported the enhanced reduction of the oxygen catalytic activity of platinized graphene/ceramics due to the better structural stability supported by the ceramic particles. Mathur *et al*.^24^ found the enhanced electrocatalytic performance of Fe_2_O_3_ nanoparticles supported on CaCu_3_Ti_4_O_12_. In view of the above considerations, ACu_3_Ti_4_O_12_ perovskites have great potential towards the ORR activity and photocatalytic activity, which will provide the next generation electrocatalyst.

Y_2/3_Cu_3_Ti_4_O_12_ (YCTO) is an isostructural material of CaCu_3_Ti_4_O_12_, but still remains relatively unexplored compared to other ACu_3_Ti_4_O_12_ (A = Ca, La, and Bi). CaCu_3_Ti_4_O_12_ has already been established as an advanced perovskite compound for visible light active photocatalyst^25, 26^. The complex covalent bonding network in CaCu_3_Ti_4_O_12_ demonstrates the excellent photocatalytic properties for the next generation photocatalyst and provides the foundation of the isostructural perovskites (YCTO) as a promising material for the electrocatalyst. The motivation of this work was to study the ORR activity of the new Y_2/3_Cu_3_Ti_4_O_12_ perovskite via rotating disk electrode (RDE) measurements^27^ as well as to establish the rapid and facile synthesis of this material (see supporting information S1). Y_2/3_Cu_3_Ti_4_O_12_ is a cubic double-perovskite with Y^+3^ and Cu^+2^ on the A site and Ti^+4^ on the B site. Both cations on the A and B sites in the crystal lattice allow considerable control of the band structure, which plays a significant role in the catalytic activity in perovskite materials. To the best of the authors’ knowledge, there is no report on the direct use of ACu_3_Ti_4_O_12_-based perovskite for the ORR in FC. Moreover, a direct transition of 1.8 eV for the visible light absorption of the YCTO ceramic has never been reported, but has great potential as the next generation ORR catalyst and as a photocatalyst.

## Results

### Synthesis of Y_2/3_Cu_3_Ti_4_O_12_

**Figure 1:**
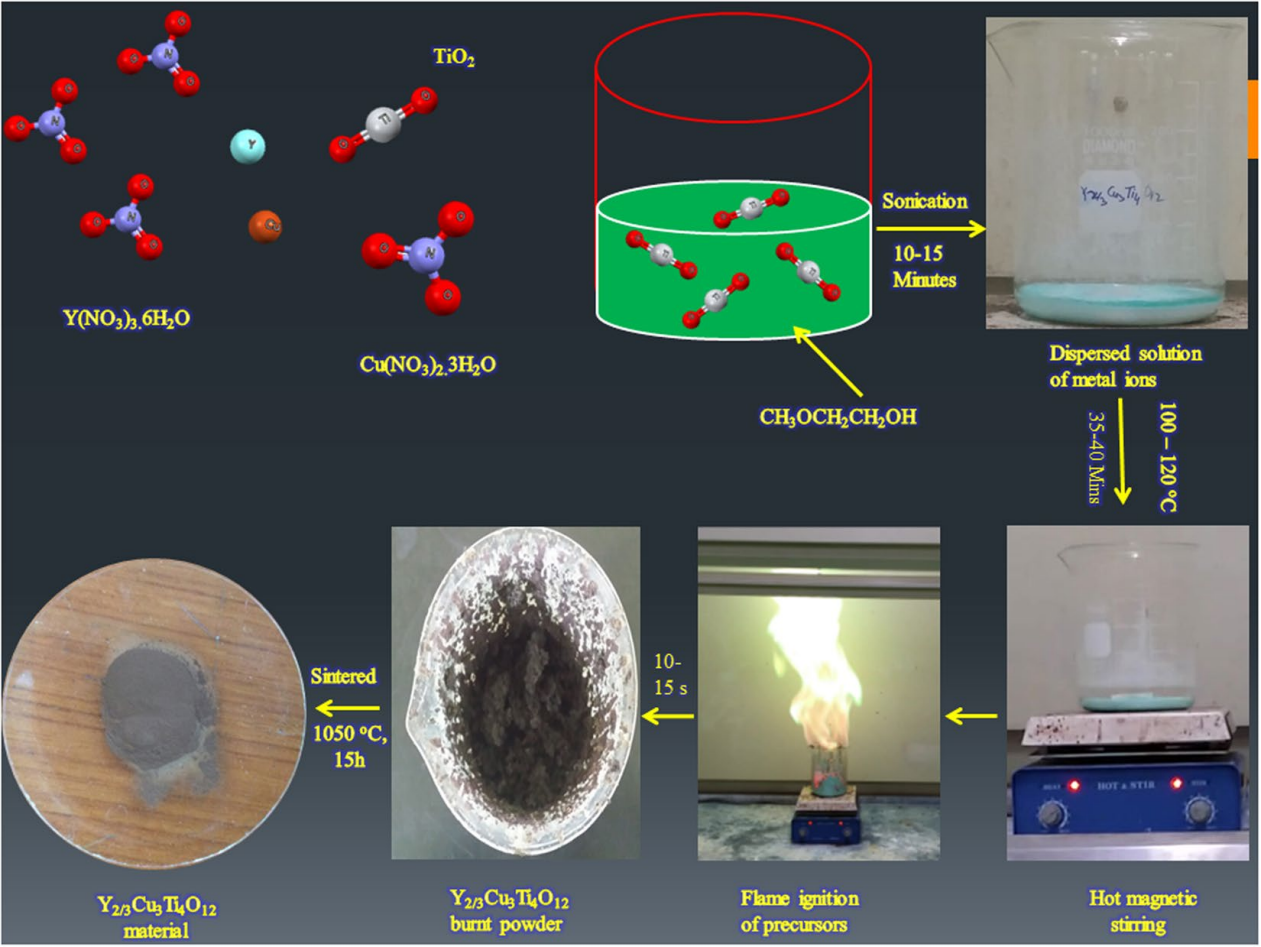
Schematic diagram of the flame synthesis procedure to obtain Y_2/3_Cu_3_Ti_4_O_12_.

Y_2/3_Cu_3_Ti_4_O_12_ perovskite was synthesized via rapid automatic flame synthesis. Supplementary information S1 highlights the merit of the current synthesis procedure over the other reported fabrication procedures for pristine Y_2/3_Cu_3_Ti_4_O_12_. Figure 1 presents a schematic diagram of the experimental procedure of automatic flame synthesis. The video and photographs during the occurrence of auto flame synthesis reaction under open air conditions is also available in the supplementary information S2 and S3.

**Figure 2:**
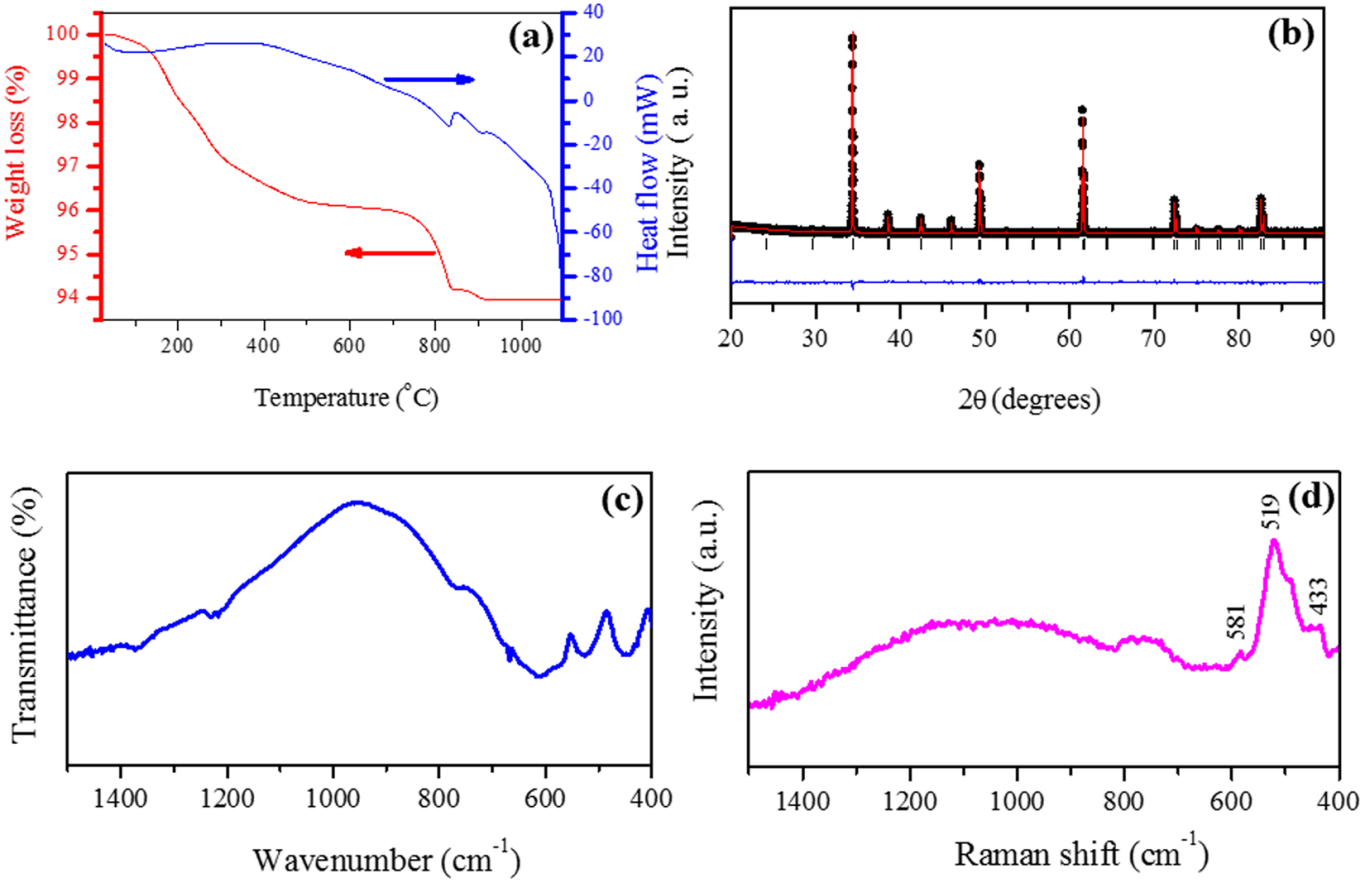
(**a**) TGA/DSC curves for the precursor powder of Y_2/3_Cu_3_Ti_4_O_12_ and (**b**) Rietveld refinement fit of Y_2/3_Cu_3_Ti_4_O_12_. Black dot, red line, black vertical bars and blue line in the figure represent the experimental data, fitted curve, Bragg’s reflections and difference profile respectively. (**c**) Fourier transforms infrared and (**d**) Raman spectra of the precursor powders heat treated at 1050 °C for 15 hrs.

Figure 2(a) shows the simultaneous TG and DSC curves during the thermal decomposition process of the auto flame-synthesized YCTO precursor powder. As shown by the TG curve, the total weight loss is approximately 5.76%. The loss of a very small weight of precursor due to the formation of crystalline phases of metal oxides (see supplementary information Fig. S1) highlights the low probability of the presence of excess organic components after the flame reaction, which is the reason to be required only one sintering step for producing a perovskite phase. The thermal decomposition process could be divided into two steps. The first step has a very small weight loss due to the evaporation of residual water and organic solvents at temperatures below 430 °C. In the second step, weight loss occurs in the temperature range of 430–850 °C accompanied by the decomposition of metal nitrates/organic groups. Above 850 °C, there is almost no weight loss in the TG curve corresponding to the endothermic peak at 848 °C, which was observed in the DSC curve. The absence of a peak in the TG curve beyond 850 °C, is also supported by DSC analysis, which confirms the formation of a YCTO perovskite phase due to a combination of crystalline oxides with solid TiO_2_ after 850 °C.

### Structural and microstructural analyses of auto flame synthesized Y_2/3_Cu_3_Ti_4_O_12_

A Rietveld refinement is also required to determine the actual structure of the auto flame synthesized YCTO powder for an approximate model structure. The full-pattern Rietveld refinement was carried out using the Fullprof program^28^. The structural parameters reported by Wong-Ng *et al*.^29^ were taken as the initial model for the refinement. In the refinements, a Pseudo-Voigt function and 6^th^ coefficient polynomials were used to define the profile shape and the background, respectively. Figure 2(b) shows the results of the Rietveld refinement, which is well matched between the observed and experimental data to ensure a single cubic perovskite phase. The refined lattice parameter in the present case was 7.37743 Å^29^, which is very close to that of a previous report, 7.37757 Å^29^.

The formation of a single cubic perovskite phase of an auto flame-synthesized YCTO powder was analyzed further with those obtained by Fourier transform infrared (FT-IR) and Raman spectroscopy, as shown in Fig. 2(c) and (d). The main FT-IR absorption peaks were found at 576, 528, and 446 cm^−1^, which were assigned to the absorption regions of the Ti ion ascribed to *ν*_Ti−O_ (653–550 cm^−1^) and *ν*_Ti−O−Ti_ (495–436 cm^−1^) as shown in Fig. 2(c) ^30^. Therefore, the main resonance absorptions are in good agreement with those of a structurally similar CCTO perovskite^31^.

Figure 2(d) presents the Raman scattering spectrum, in which three main peaks are observed in the wave region of 400–600 cm^−1^. The Raman peaks at 433 and 519 cm^−1^ have the A_g_ symmetry of TiO_6_ rotation. The peak at 581 cm^−1^ was assigned to the mode of F_g_ symmetry of O–Ti–O anti-stretching. All three peaks assigned in the Raman spectrum have also been reported for isostructural CCTO perovskite^31^.

**Figure 3:**
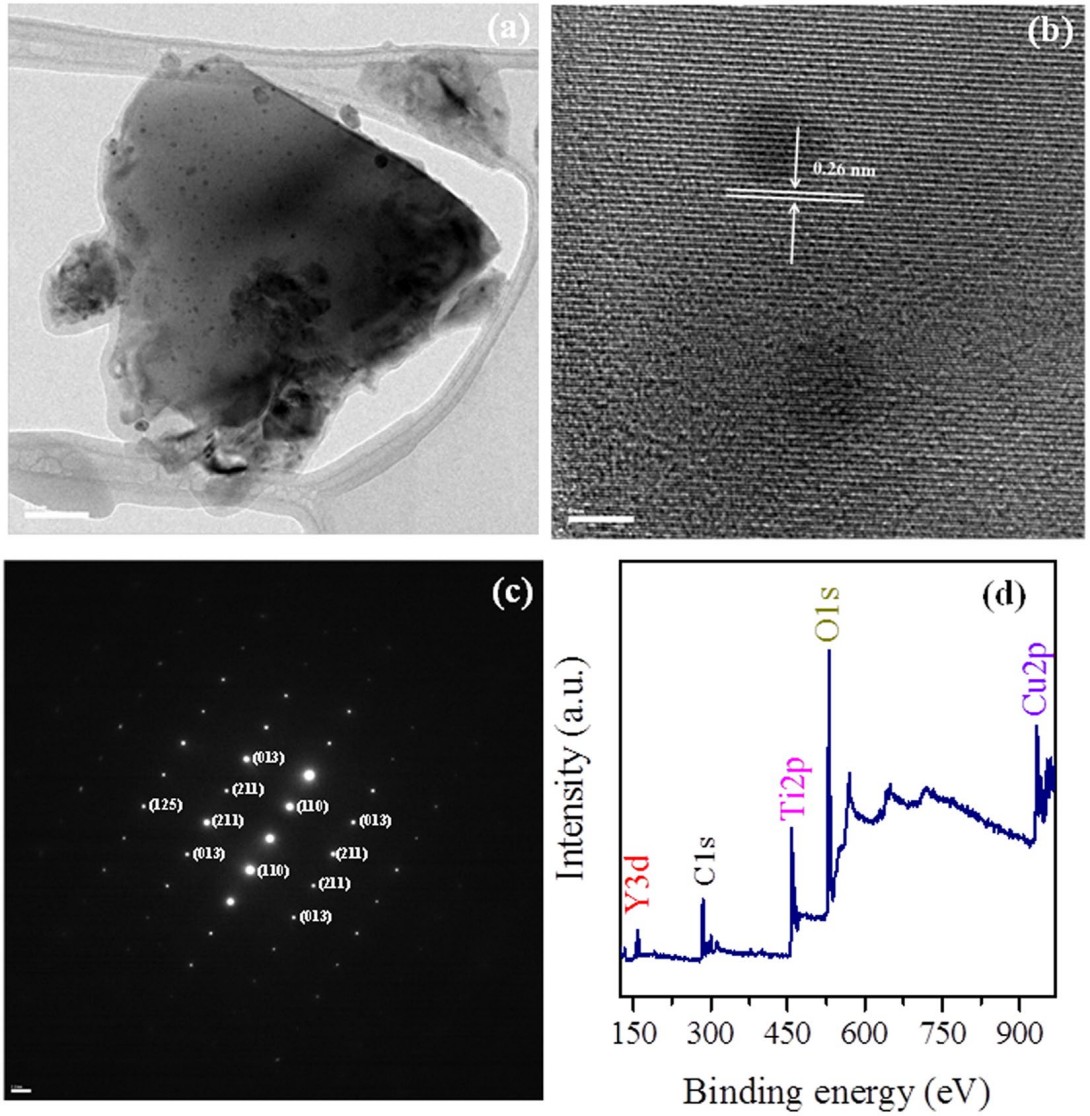
(**a**) TEM image, (**b**) HRTEM lattice image, (**c**) SAED pattern, and (**d**) XPS survey spectrum of phase-pure YCTO.

High resolution transmission electronic microscopy (HRTEM) and selected area electron diffraction (SAED) of the auto flame synthesized YCTO powder were carried out to examine the crystallinity and phase-purity further. Figure 3(a) shows a TEM image of a YCTO particle. The HRTEM image of YCTO in Fig. 3(b) shows a typical lattice fringe indicating well-crystallized YCTO particles with interplanar spacing of 0.26 nm, corresponding to the (220) crystal planes and agreed well with the XRD data for the cubic perovskite phase. The SAED pattern as shown in Fig. 3(c) has a highly symmetrical dotted lattice, which supports the highly crystalline nature of YCTO. The diffraction spots of the SAED pattern were indexed to the (211), (013), (110), and (125) reflections of the cubic phase with space group *Im*3. X-ray photoelectron spectroscopy (XPS) was carried out to determine the compositions and oxidation state of the elements present in the YCTO powder and estimate the quality of material preparation. The detailed oxidation states with the XPS fitting parameter of each element are given in supplementary information S4. As shown in Fig. 3(d), the XPS survey spectrum showed the elemental peaks of Y, Cu, Ti, and O, which is consistent with the results of EDX characterization (see supplementary information S5). The unavoidable existence of a carbon peak (C 1 s) is due to adventitious carbon species.

### Characterization by cyclic voltammetry

**Figure 4:**
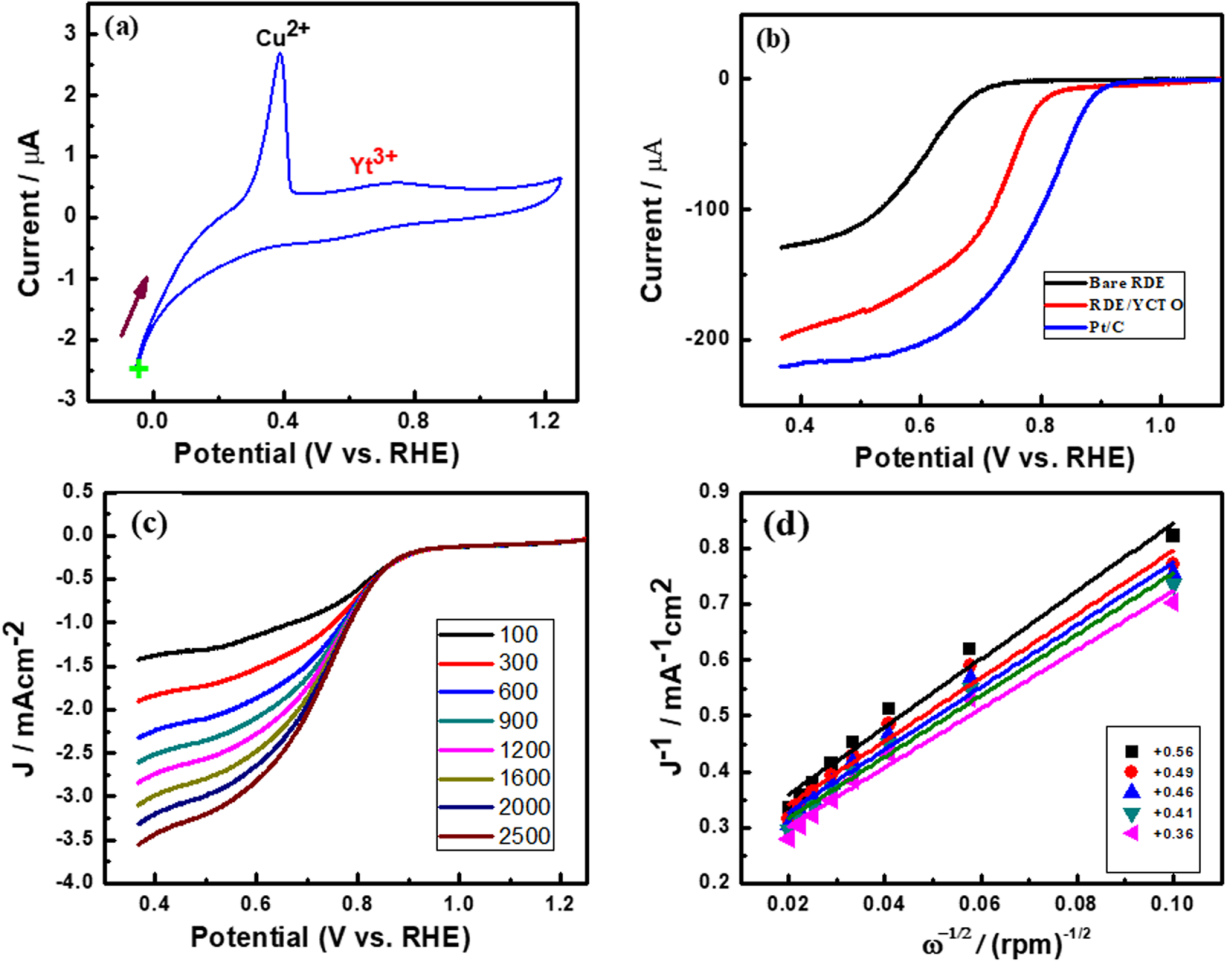
(**a**) Cyclic voltammogram of GC/Cu-Y in 0.1 M H_2_SO_4_ at a scan rate of 20 mV s^−1^. The initial point of the scan is indicated by + sign. (**b**) Linear sweep voltammograms of bare RDE and RDE/Cu-Y and RDE/Pt/C at constant rotation speed in 0.1 M KOH at 10 mV s^−1^ scan rate. (**c**) LSVs of the GC/Cu-Y electrode in O_2_-saturated 0.1 M KOH solution at a scan rate of 10 mV s^−1^ using RDE with different rotation (100, 300, 600, 900, 1200, 1600, 2000, and 2500 rpm). (**d**) Corresponding K-L plots of the ORR at different potentials, +0.56, +0.49, +0.46, +0.41, and +0.36 V.

Figure 4(a) shows the cyclic voltammogram of the prepared composite material immobilized onto the GC electrode (GC/YCTO) in 0.1 M H_2_SO_4_, in which clear oxidation peaks were observed for Cu^2+^ and Y^3+^. A clear reduction peak was observed for Y, whereas no reduction peak was observed for Cu. According to a literature survey, this is the first report of the electrochemical properties of the prepared composite material. A detailed electrochemical study was performed to obtain greater insight.

### Electrocatalytic reduction of oxygen

Figure 4(b) shows the linear sweep voltammogram of bare RDE and RDE/YCTO and RDE/Pt/C electrode at constant rotation speed in an oxygen saturated 0.1 M KOH solution at 10 mV s^−1^ scan rate. At bare RDE reduction of oxygen begins at +0.71 V, whereas at RDE/YCTO and Pt/C it begins at +0.84 and 0.93 V respectively. Thus, reduction of oxygen at RDE/YCTO takes place 130 mV more positive and 90 mV less positive as compared to bare RDE and Pt/C respectively. Current density at RDE/YCTO is approximately 2 times higher as compared to bare RDE but less than Pt/C. This shows that the active sites on prepared composite materials are not well-defined as those on the Pt surface.

To examine the electron transfer kinetics of GC/YCTO during O_2_ reduction, a hydrodynamic voltammetry study was carried out using RDE in an O_2_ saturated 0.1 M KOH solution at different rotation speeds from 100 to 2,500 rpm, as shown in Fig. 4(c). The linear sweep voltammograms were recorded in an O_2_ saturated 0.1 M KOH electrolyte at a scan rate of 10 mV s^−1^ using a RDE. The measured current intensities for the modified electrode showed improvement with increasing rotating rate due to the enhanced diffusion of oxygen from solution to the electrode surface. In contrast, the LSVs of the GC/YCTO electrode revealed a direct four-electron transfer pathway for O_2_ reduction. Very few reports are available for O_2_ reduction using Cu as a catalyst, and there are no reports of YCTO for O_2_ reduction.

Figure 4(d) shows the Koutecky-Levich plots of J^−1^ vs. ω^−1/2^ at various potentials to determine the kinetic parameters of GC/YCTO. The kinetic parameters were calculated based on the Koutecky-Levich equations. The number of electrons transferred (n) in the ORR process for GC/YCTO electrode was calculated using the following Koutecky-Levich equation (1) ^32^

1 $$\begin{array}{c}\frac{1}{{\bf{J}}}=\frac{1}{{{\bf{J}}}_{{\bf{K}}}}+\frac{1}{{\bf{B}}{{\boldsymbol{\omega }}}^{1/2}}\\ {\boldsymbol{B}}={\bf{0.62}}\,{\bf{nF}}{{\boldsymbol{D}}}_{{\boldsymbol{O}}{\bf{2}}}^{2{\boldsymbol{/}}3}{{\boldsymbol{\nu }}}^{-1{\boldsymbol{/}}6}{{\bf{C}}}_{{\boldsymbol{O}}{\bf{2}}}\end{array}$$

**Figure 5:**
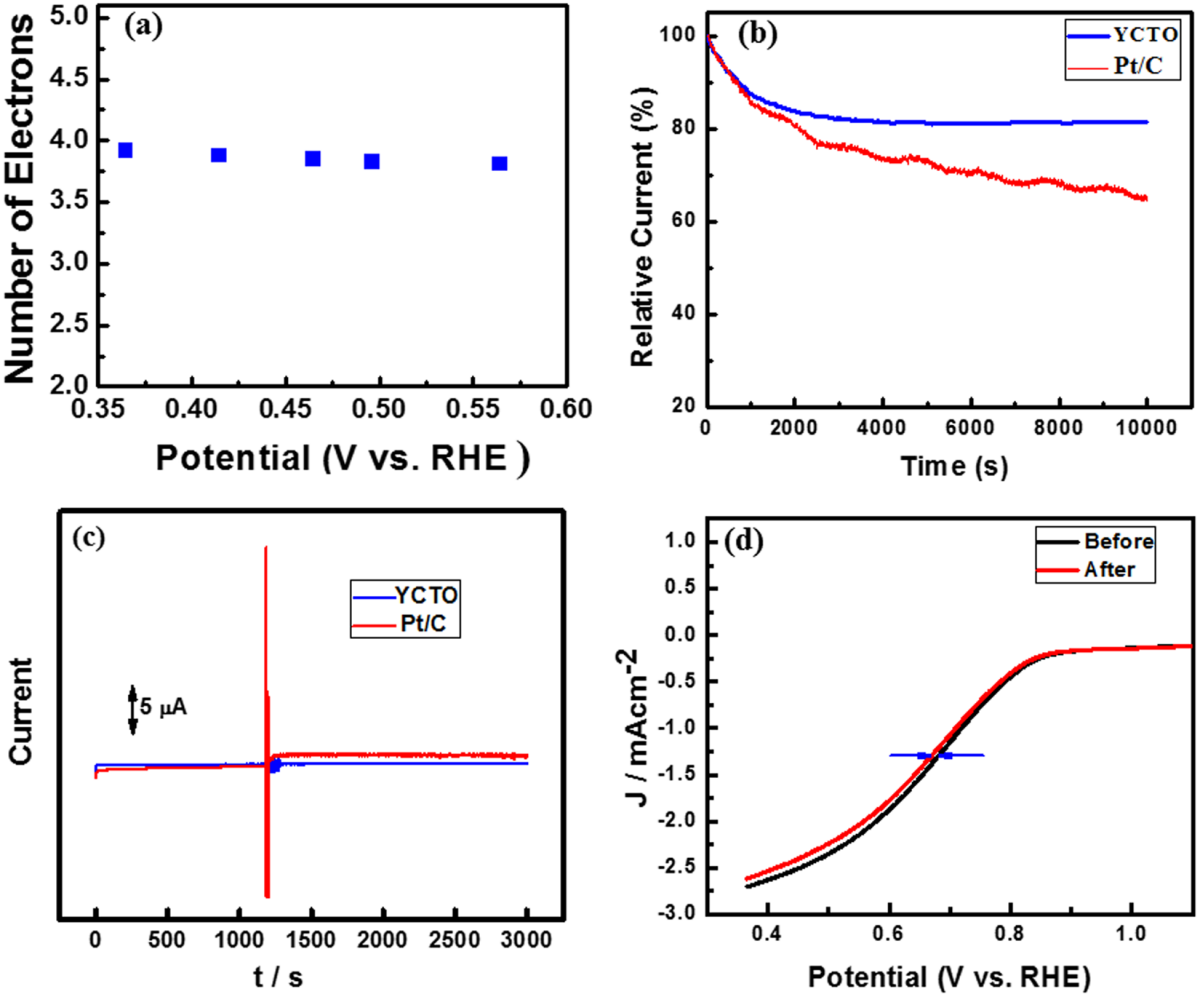
(**a**) Number of electrons transferred vs. the potential as calculated from the KL plots. (**b**) Relative current vs. time responses at −0.2 V in O_2_-saturated 0.1 M KOH solution for GC/Cu-Y and 20 wt.% GC/Pt/C electrodes. (**c**) Chronoamperometric response of GC/Cu-Y and Pt/C at +0.60 V in oxygen saturated 0.1 M KOH followed by the addition of 3.0 M methanol. (**d**) Linear sweep curves of the ORR on the GC/Cu-Y electrode before and after 10,000 cycles in O_2_-saturated 0.1 M KOH at 25 °C.

Where F is the Faraday constant (96,500 C mol^−1^), D_O2_ is the diffusion coefficient of O_2_ in a 0.1 M KOH aqueous solution (1.9 × 10^−5^ cm^2^ s^−1^), ν is the kinematic viscosity of the solution (1.0 × 10^−2^ cm^2^ s^−1^), and C_O2_ is the oxygen concentration (1.2 × 10^−6^ M) in O_2_ saturated solutions (0.1 M KOH). The slopes of the corresponding Koutecky-Levich plots (J^−1^ *vs*. ω^−1/2^) at different reduction potentials are almost parallel and close to that of the calculated line for the four-electron reduction of oxygen in Fig. 5(a). The non-zero intercepts of the extrapolated Koutecky-Levich lines suggest that the O_2_ reduction process on GC/YCTO is under mixed kinetic-diffusion control in a large potential range. The linearity and parallelism of the plots are considered typical of first-order reaction kinetics with respect to the concentration of dissolved O_2_. As shown in Fig. 4(d), the Koutecky-Levich plots of J^−1^ vs. ω^−1/2^ at potentials of +0.56, +0.49, +0.46, +0.41, and +0.36 V on GC/YCTO showed good linearity. From the slope (1/B) of the Koutecky-Levich plots, the n values for GC/YCTO at various potentials were calculated according to the above mentioned formula and were very close to four. Figure 5(a) shows the corresponding plots of the n values versus the potential. The number of electrons involved in the ORR was close to 4 at all potentials, indicating that reduction proceeds predominantly through a kinetically favorable pathway of direct conversion from O_2_ to OH^−^, as reported for noble metals, such as Pt, Pd, Ag-Au alloys, etc. refs 33, 34. As most of these types of ceramic materials are used in solid oxide fuel cells, the total number of electrons involved and the onset potential of the prepared composite is comparable to previously studies, as listed in Table 1 ^35–48^. There are very few reports are available for such kinds of material synthesized via sol-gel technique. Hu *et al*. prepared La_1−x_Ca_x_MnO_3_ perovskite-graphene composites and used for oxygen reduction^49^. However, they have neither studied the stability and poisoning of the material nor compared the performance with commercially available Pt/C catalysts.

**Table 1:** Comparison of the ORR performance of the electrocatalyst reported earlier.

Catalysts	Onset Potential (V) RHE	Peak Potential(V)	N	References
N-doped carbon frameworks	0.79	—	3.95	35
N-doped hollow fibers	0.75	0.77	3.6	36
ZIF-derived porous carbon	0.83	0.68	3.3	37
sulphur-doped graphene	0.88	0.69	3.13	38
N-CNT frameworks	0.97	0.87	3.97	39
Ordered mesoporous carbon	0.81	—	3.1	40
Co_3_O_4_/graphene Sheets	0.88	0.84	3.9	41
Co,N-CNF	0.882	—	—	42
Co/graphene Sheets	0.80	—	—	43
LaCu_0.5_Mn_0.5_O_3_	0.80	—	≈4	44
La_0.5_Sr_0.5_CoO_2.91_	≈0.75	—	3.1	45
BaMnO_3_	−0.19 (Ag/AgCl)	—	3.4–3.7	46
LaFe_0.95_Pd_0.05_O_3_	≈−0.2 (Ag/AgCl)		3.94	47
La_0.5_Sr_0.5_Co_0.8_Fe_0.2_O_3_	≈0.9		3.9–4	48
Y_2/3_Cu_3_Ti_4_O_12_	0.84	0.7	≈4	Present Work

Durability and tolerance is another important aspect of fuel cell catalysts that must be considered during the construction of a fuel cell. The chronoamperometric measurements of GC/YCTO and GC/Pt/C were carried out at 0.6 V for 10,000 s, as shown in Fig. 5(b). The 20% current loss in the initial current for GC/YCTO was observed, while approximately 36% loss of the initial current for GC/Pt/C was noted. This indicates that the GC/YCTO catalyst has much better durability than the commercial available Pt/C. The obtained stability is comparable to previously reported literature^50–52^. The GC/YCTO electrode was subjected to further testing to determine the possible crossover and stability toward the ORR. To examine the possible crossover effect in the presence of other fuel molecules (e.g., methanol), the current vs. time chronoamperometric responses for the ORR at the GC/YCTO and GC/Pt/C electrodes were measured, as shown in Fig. 5(c). A sharp positive and negative current change was observed for the Pt/C electrode upon the addition of 3.0 M methanol. On the other hand, the corresponding amperometric response for the GC/YCTO electrode remained almost unchanged even after the addition of methanol. This clearly indicates that the GC/YCTO electrocatalyst has higher fuel selectivity toward the ORR than the commercial Pt/C electrocatalyst.

The electrochemical stability of the ORR electrocatalyst depends on the structural stability of the material. To gain further insight into the stability of the catalytic activity of the GC/YCTO electrocatalyst in the present investigation, linear sweep voltammetry was performed between potential windows of 0.36 to 1.36 V at a 10 mV s^−1^ scan rate under oxygen purging. Figure 5(d) presents the linear sweep voltammograms of the GC/YCTO electrode before and after 10,000 continuous cycles in an O_2_ saturated 0.1 M KOH solution at room temperature. After 10,000 continuous potential cycles, the half-wave potential E_1/2_ of YCTO exhibited a negative shift of only 9 mV in Fig. 5(d), which is much lower than previously reported electrocatalysts for ORR. The changes in E_1/2_ of various electrocatalyst after several cycles have been listed in Table 2 ^43, 53–56^. The current did not show a significant decrease after 10,000 continuous cycles, which demonstrates the excellent stability of the GC/YCTO electrode for ORR applications than the commercial Pt/C electrocatalyst. SEM images of YCTO coated on the GC disk were recorded before and after the stability test, but no distinguishable morphological change was observed (see supplementary information S6).

**Table 2:** Comparison of the E_1/2_ of various electrocatalyst for oxygen reduction reaction.

Electrocatalyst	Number of Cycles	Changes in E_1/2_ (mV)	References
P-Z8-Te-1000	8000	11	53
C-N-Co	10000	9	54
Co, N-CNF	5000	10	55
NT-G	8000	11	56
PANI-Fe-C	10000	10	57
Y_2/3_Cu_3_Ti_4_O_12_	10000	9	Present Work

## Discussion

Pristine Y_2/3_Cu_3_Ti_4_O_12_ powder was synthesized in a single step at 950 °C for 15 h, which is relatively simple, rapid with short sintering duration as compared to other conventional synthesis routes. The confirmation of phase formation and the composition by different physiochemical characterization techniques such as XRD, IR, Raman, XPS, and EDX showed the fabrication of good quality of YCTO material. The present automatic flame synthesis procedure is rapid and facile for Y_2/3_Cu_3_Ti_4_O_12_ with the merits of simplicity, energy, and time saving. In future, it may be considered for the large scale production of isostructural ACu_3_Ti_4_O_12_ (A = Cd, Sm_2/3_, Bi_2/3_, and La_2/3_) perovskite. The electrochemical RDE analysis showed that the number of electrons transferred in ORR is close to 4, indicating that Y_2/3_Cu_3_Ti_4_O_12_ promotes the four electron transfer pathway. This study clearly showed that Y_2/3_Cu_3_Ti_4_O_12_ is a promising material as an excellent electrocatalyst with enhanced fuel tolerance selectivity, durability, and electrochemical stability as well as higher ORR activity compared to commercial Pt/C. In addition, the UV-visible study of YCTO revealed a direct transition at 1.8 eV for the visible light absorption, which should be considered as a third generation photocatalyst similar to the well-established isostructural CaCu_3_Ti_4_O_12_ perovskite reported earlier. The energy band gap (E_g_) is an important feature of ABO_3_ type metal oxides, which determine their applications in photocatalysis, electrocatalytic activity, and optoelectronics. For this purpose, the optical characterization was carried out (see supplementary information S7) and supported the results of the electrocatalytic activity of YCTO. A direct transition with an energy band gap (E_g_) of 1.8 eV was determined from the UV-visible diffuse reflectance spectrum. Furthermore, the electrocatalytic ORR activity and photocatalytic of the Y_2/3_Cu_3_Ti_4_O_12_ perovskite were correlated with the covalent networking and broad diversity of A- and B-site transition metals^22^. The electrocatalytic activities of ABO_3_-type perovskite are associated with the corner shared network of TiO_6_ octahedron, which can facilitate electron transfer and oxygen transfer in the lattice^57^. In addition, the large atoms at the A-site of ABO_3_ allow stabilization of the multiple valence states of B-site metal cation^58^. Overall, YCTO has great potential for the next generation ORR catalyst and photocatalyst.

## Methods

### Material synthesis

Y(NO_3_)_3_·6H_2_O (99.80%, Aldrich), Cu(NO_3_)_2_·3H_2_O (99.0%, Junsei), TiO_2_ (99.9%, Sigma Aldrich), and 2-methoxyethanol (99.0%, Alfa Aesar) were used to prepare the Y_2/3_Cu_3_Ti_4_O_12_ (YCTO). A stoichiometric amount of Y(NO_3_)_3_·6H_2_O and Cu(NO_3_)_2_·3H_2_O was dissolved in a minimum amount of 2-methoxyethanol. A stoichiometric amount of solid TiO_2_ was added to the homogeneous metal nitrate solution of Y^3+^ and Cu^2+^, and sonicated for 10–15 min. The heterogeneous mixture of metal ions was formed and heated to 90–120 °C for 30–35 min on a hot plate using a magnetic stirrer to evaporate the organic solvent until self-ignition took place, which exhausted a large amount of gases with a greenish flame, and produced a fluffy mass of YCTO precursor powder within 15–20 s. Upon cooling, the resulting product was ground using pestle and mortar and sintered without a pre-calcination step at 950 and 1050 °C for 15 hrs in an electrical furnace.

### Material characterization

Thermogravimetric (TG) and differential scanning calorimetry (DSC) measurements of the auto flame synthesized precursor powder were carried out on SDT Q600 thermal analyzer (TA Instruments) from room temperature to 1050 °C with 10 °C min^−1^ in a nitrogen atmosphere. The crystalline structure of the precursor powder and sintered at 950 and 1050 °C for 15 hrs was determined by X-ray diffraction (XRD, Rigaku Ultima IV, Japan) using Cu Kα radiation. The crystallinity and composition of the YCTO powder were investigated by transmission electron microscopy (TEM, JEM-2100F, Jeol, Japan) and scanning electron microscopy (SEM, JEOL JSM6500F, Japan) attached to energy-dispersive X-ray spectroscopy analyzer for elemental analysis. X-ray photoelectron spectroscopy (XPS, Thermo Fisher Kα, USA) was performed to determine the oxidation state and composition of the synthesized material. The Fourier transform infrared (FT-IR, Shimadzu IRAffinity-1S, Japan) spectrum of YCTO was acquired using KBr pellets at a suitable composition from 400 to 1600 cm^−1^. Raman spectra (resolution: 4 cm^−1^) were collected by a Raman microscope equipped with a diode laser (λ_ex_ = 785 nm, power = 44 mW), a CCD detector, and a holographic grating (Kaiser Optical Inc., Ann Arbor, MI, USA). Each sample was positioned on a microscope stage and the laser beam was focused with an objective lens (10×/0.25 NA) to collect spectra.

### Electrochemical Measurements

#### Instrumentation

The electrochemical experiments were performed with a CHI-660C (CH Instruments, USA) using a three-electrode system with GC (CH Instruments, area = 0.07 cm^2^) or modified GC as working electrode, platinum wire as the counter electrode and Ag/AgCl as the reference electrode. Rotating disk electrode (RDE) voltammetry was conducted on a Pine Research Instrument (AFMSRCE, USA) modulated speed rotator. All electrochemical experiments were carried out at 20 °C, and the potentials were referenced to Ag/AgCl. The solutions were purged with N_2_ or O_2_ for 20–30 min before the electrochemical experiments. All potentials reported were referenced to the reversible hydrogen electrode (RHE) through a RHE calibration. Eq. 1 was used to convert the obtained potential (vs. Ag/AgCl) to the RHE.

1 $$\begin{array}{c}{{\rm{E}}}_{{\rm{RHE}}}={{\rm{E}}}_{{\rm{Ag}}/{\rm{AgCl}}}+0.059\,{\rm{pH}}+{{{\rm{E}}}_{{\rm{Ag}}/{\rm{AgCl}}}}^{0}\\ \quad \quad \,\,\,\,\,({{{\rm{E}}}_{{\rm{Ag}}/{\rm{AgCl}}}}^{0}=+0.199\,{\rm{V}})\end{array}$$

### Preparation of Electrode (GC/Cu-Y)

Typically, 2 mg of the Cu-Y composite was dissolved in 1.0 mL of ethanol and sonicated for 30 min. Using a micropipette, 10 µL of this solution was dropped to coat onto the surface of the GC electrode and 20 µL at RDE, respectively, and dried for 30 min.

## Electronic supplementary material

Supplementary Information

Supplementary Information S2
