# Supplementary material for: Yttrium Copper Titanate as a Highly Efficient Electrocatalyst for Oxygen Reduction Reaction in Fuel Cells, Synthesized via Ultrafast Automatic Flame Technique
Source: Sci Rep. 2017 Aug 24;7:9407. doi: 10.1038/s41598-017-09661-9 (PMC5571221; doi:10.1038/s41598-017-09661-9)
Supplement: Supplementary file 1 — Supplementary Information [file 41598_2017_9661_MOESM1_ESM.pdf]

Supplementary Information

Yttrium Copper Titanate as a Highly Efficient Electrocatalyst for Oxygen Reduction Reaction in Fuel Cells, Synthesized via Ultrafast Automatic Flame Technique

Laxman Singh[a], Uday Pratap Azad[b], Satendra Pal Singh[c], Vellaichamy Ganesan[b], U.S. Rai[b] and Youngil Lee*[a]

[a]Department of Chemistry, University of Ulsan, 93 Daehak-ro Nam-gu, Ulsan 44610, Republic of Korea

[b]Department of Chemistry, Institute of Science, Banaras Hindu University,Varanasi – 221005, Uttar Pradesh, India

[c]Faculty of Nanotechnology and Advanced Materials Engineering, Sejong University, Seoul 05006, Republic of Korea

Supplementary Information S1: $Y_{2/3}Cu_3Ti_4O_{12}$ perovskite is a potential material for electronic industries for electronic devices, electro-catalysts, and other industrial applications. The physical and chemical properties of ABO_3 perovskite are influenced directly by the processing routes. Very few studies have examined the synthesis of pristine $Y_{2/3}Cu_3Ti_4O_{12}$ perovskite in comparison with structurally similar CCTO. The pure cubic phase of pristine $Y_{2/3}Cu_3Ti_4O_{12}$ is generally synthesized by a solid-state technique using the metal oxides of Y_2O_3, CuO, and TiO_2, which require tedious work with high temperature sintering for long durations with repeated intermediate grinding[1]. As reported in the literature, the sol-gel processing[2] and combustion synthesis[3] of pristine $Y_{2/3}Cu_3Ti_4O_{12}$ required a multistep, complex procedure, and took a long time during the synthesis procedure along with some secondary phases of TiO_2, and CuO and

$CaTiO_3$ formed[3]. Table S1 compares the precursor and synthetic condition used in the synthesis of pristine $Y_{2/3}Cu_3Ti_4O_{12}$ by different routes. In previous work based on chemical synthesis, the impurity phases were only observed in the pristine YCTO, while a single cubic phase in YCTO was obtained in most metal cation-substituted YCTO materials. The incorporation of metal cations into the pristine $Y_{2/3}Cu_3Ti_4O_{12}$ stimulates the formation of a cubic phase and controls metal ions diffusion during the sintering process[4]. The single cubic phase of pristine $Y_{2/3}Cu_3Ti_4O_{12}$ was formed via direct sintering at 950 °C for 15 hrs (see supplementary Fig. S1). The precursor powder immediately after the flame reaction at room temperature also showed the crystalline phase of metal oxides with a minor phase of YCTO, which revealed better results than the other oxidant fuel-based combustion synthesis requiring multistep and a long duration. The current automatic flame synthesis procedure is revealed the feasibility to the fabrication of other isostructural perovskite.

Supplementary Information S2:

VideoS2: Video of automatic flame synthesis reaction took place in an open air condition to obtain the $Y_{2/3}Cu_3Ti_4O_{12}$ precursor. Flame ignition of the metal nitrate of yttrium and copper along with solid $TiO_2$was completed within few seconds (15–20 s). This is a highly exothermic flame reaction. The entire synthetic procedure to obtain $Y_{2/3}Cu_3Ti_4O_{12}$ from start to finish was complete within a few minutes.

Supplementary Information S3:

Photographs were taken step-by-step during the automatic flame synthesis reaction of $Y_{2/3}Cu_3Ti_4O_{12}$.

Supplementary Information S4:

X-ray photoelectron spectroscopy (XPS) was used to identify the chemical nature of the surface of $Y_{2/3}Cu_3Ti_4O_{12}$. Fig. S4 shows the spectral regions of Y 3d, Cu 2p, Ti 2p, and O 1s transitions. In the XPS spectrum of Y3d as shown in Fig. S4(a), two main peaks were observed at 157.6 and 159.4 eV, which were assigned to $Y3d_{5/2}$ and $Y3d_{3/2}$, respectively. In Fig. S4(b), the well fitted Cu 2p transitions were observed at 933.6 and 953.4 eV with satellite peaks indicating the existence of Cu as Cu^{2+}/Cu^{3+}. XPS spectrum of Ti 2p in Fig. S4(c) shows a binding energy (B. E.) between 455 – 465 eV with two prominent peaks at 458.2 eV and 463.8 eV corresponding to Ti $2p_{3/2}$ and Ti $2p_{1/2}$, respectively[5]. The high-resolution spectrum of O 1s is shown in Fig. S4(d) which displayed the single symmetric peak positioned at 529.5 eV, and the B. E. value is well agreement with the XPS spectra of oxygen position in ABO_3 type perovskite[6]. The XPS measurements of YCTO confirmed that the oxidation state of Y is +3, Cu is Cu^{2+} /Cu^{3+}, and Ti^{4+} is +4. This is also supported by the CV results.

Supplementary Information S5:

The energy dispersive X-rays (EDX) and line scan spectra of the powder sample for pristine $Y_{2/3}Cu_3Ti_4O_{12}$ were carried out to detect the percentage of elements and their uniform distribution inside the material. The EDX spectrum in Fig. S5(a) clearly shows the presence of Y, Cu, Ti, and O with atomic percentages of 2.41, 11.59, 18.17, and 67.83 %, respectively. The Cu/Y ratio was 4.8. EDX analysis is shown the formation of pristine $Y_{2/3}Cu_3Ti_4O_{12}$ approaching stoichiometry without impurities. Fig. S5(b-e) shows the high resolution EDX mapping images of Y, Cu, Ti, and O, which reveal a uniform distribution of all elements in pristine $Y_{2/3}Cu_3Ti_4O_{12}$. Furthermore, the EDX line-scan profiles of YCTO material have also been carried out as shown in Fig. S5(g), which also show the existence and uniform distribution of all of Y, Cu, Ti and O

elements in the YCTO material. This confirmed that the present auto-flame synthesis is a facile and practical process for obtaining highly pure pristine $Y_{2/3}Cu_3Ti_4O_{12}$.

Supplementary Information S6:

The SEM analysis has been carried out before and after electrochemical measurement (see Fig. S6). SEM images of YCTO coated on the GC disk were recorded before as shown in Fig. S6(a) and after the stability test as shown in Fig. S6(b). Even after 10,000 cycles at 10 mV s^{-1}, there is no distinguishable morphological change was observed, which further confirms their structural robustness of YCTO against repeated cyclability test.

Supplementary Information S7:

Fig. S7(a) shows typical UV-visible diffuse reactance spectrum of YCTO powder, which has two broad bands at approximately 300–600 and 600-700 nm. The optical bands between 600-700 nm suggest the strong optical property in the visible region. The optical energy band gap was estimated from the $[F(R)\times h\nu]^{1/n}$ versus E plot (n = ½ for direct transition), as shown in Fig. S7(b). A direct transition of 1.8 eV was calculated from the tangent line in the plot and suggests that the YCTO material has an appropriate bandgap for photocatalytic activity under visible light irradiation. The band gap for YCTO is comparable to that reported for isostructural $CaCu_3Ti_4O_{12}$. The optical study also supports the efficient electrocatalytic activity of YCTO in fuel cells.

References:

(1) Liang, P.; Yang, Z.; Chao, X.; Liu, Z. Giant dielectric constant and good temperature stability in $Y_{2/3}Cu_3Ti_4O_{12}$ceramics. *J. Am. Ceram. Soc.* **95**, 2218–2225 (2012).

(2) Li, J.; Liang, P.; Yi, J.; Chao, X.; Yang, Z.; Phase formation and enhanced dielectric response of $Y_{2/3}Cu_3Ti_4O_{12}$ ceramics derived from the sol–gel process. *J.Am.Ceram.Soc.* **98**, 795–803 (2015).

(3) Sharma, S.; Yadav, S. S.; Singh, M. M.; Mandal, K. D. Impedance spectroscopic and dielectric properties of nanosized $Y_{2/3}Cu_3Ti_4O_{12}$ ceramic. *J. adv. dielect.* **4**, 1450030-1450038 (2014).

(4) Jumpatam, J.; Mooltang, A.; Putasaeng, B.; Kidkhunthod, P.; Chanlek, N.; Thongbai, P.; Maensiri, S.; Effects of Mg^{2+} doping ions on giant dielectric properties and electrical responses of $Na_{½}Y_{½}Cu_3Ti_4O_{12}$ ceramics. *Ceram. Int.* **42**, 16287–16295 (2016).

(5) Singh, L.; Kim, I. W.; Woo, W. S.; Sin, B. C.; Lee, H.; Lee, Y.; A novel low cost non-aqueous chemical route for giant dielectric constant $CaCu_3Ti_4O_{12}$ ceramic. *Solid State Sci.* **43**, 35–45 (2015).

(6) Singh, L.; Kim, I. W.; Sin, B. C.; Rai, U. S.; Hyun, S. H.; Lee, Y.; Combustion synthesis of nanostructured $Ba_{0.8}(Ca,Sr)_{0.2}TiO_3$ ceramics and their dielectric properties. *Ceram. Int.* **l41**, 12218–12228 (2015).

Table S1: Precursor and synthetic condition used to fabricate the $Y_{2/3}Cu_3Ti_4O_{12}$ by different routes

**Y precursor**	**Cu precursor**	**Ti precursor**	**Synthesis route**	**Processing duration for the YCTO precursor powder**	**Calcination Temperature/duration**	**Sintering Temperature/duration**	**Ref.**
Y_2O_3	CuO	TiO_2	solid state	Ball mill (10 h + 10 h)	900-970 °C/ 10 h	1060 °C/25 h	1
$Y(NO_3)_3 \cdot 6H_2O$	$Cu(NO_3)_2 \cdot 3H_2O$	$Ti(OC_4H_9)_4$	sol-gel	Multistep duration (?) + aging gel (8 h) + drying gel (?)	650-850 °C/ 10 h + 910 °C/10 h	1040-1060 °C/25 h	2
$Y(NO_3)_3 \cdot 6H_2O$	$Cu(NO_3)_2 \cdot 3H_2O$	TiO_2	Combustion synthesis	Multistep duration (?) + dry gel (?) ~48 h	500-800 °C/ 8h	950 °C/12 h	3
$Y(NO_3)_3 \cdot 6H_2O$	$Cu(NO_3)_2 \cdot 3H_2O$	TiO_2	automatic flame synthesis	45-50 mins	-	950 °C/15 h	This work

Fig. S1

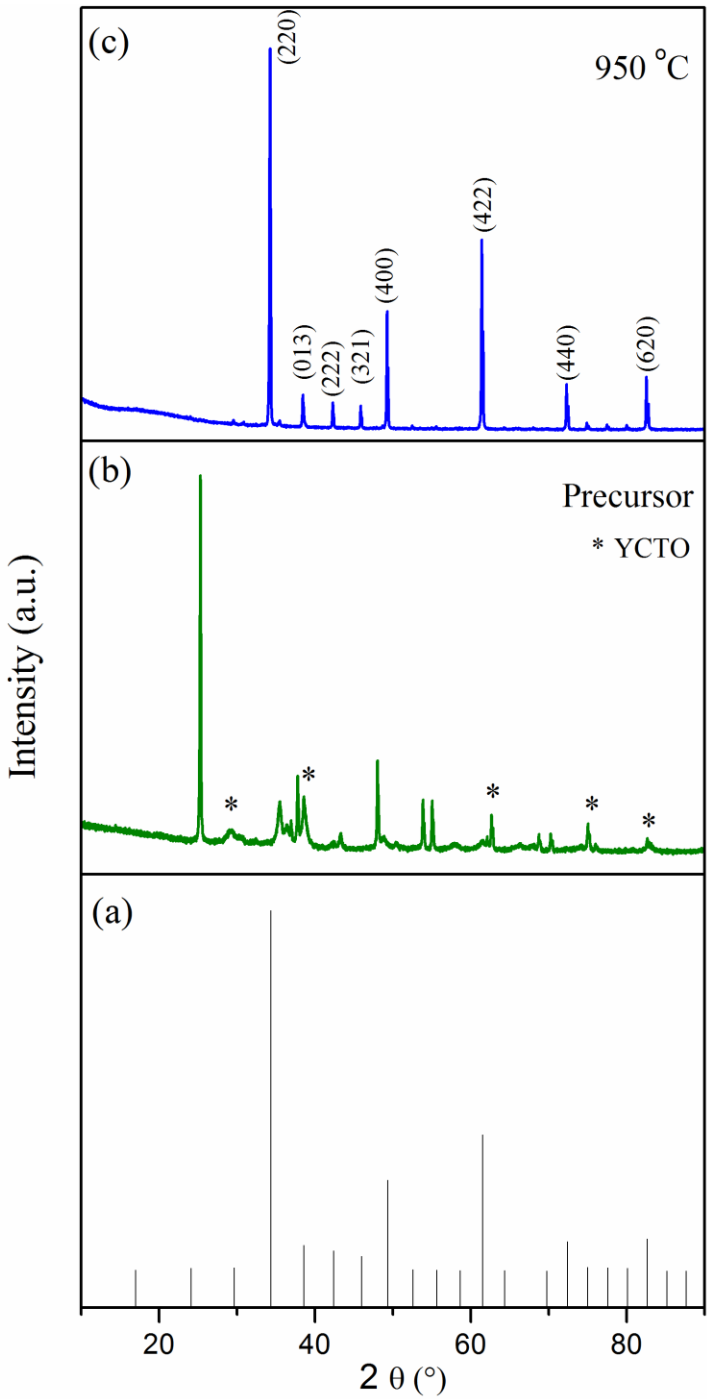

Fig. S1 XRD patterns of pristine $Y_{2/3}Cu_3Ti_4O_{12}$ for (a) reference, (b) precursor powder obtained after completion of auto flame reaction, and (c) direct sintered powder at 950 °C for 15 hrs.

Fig. S3

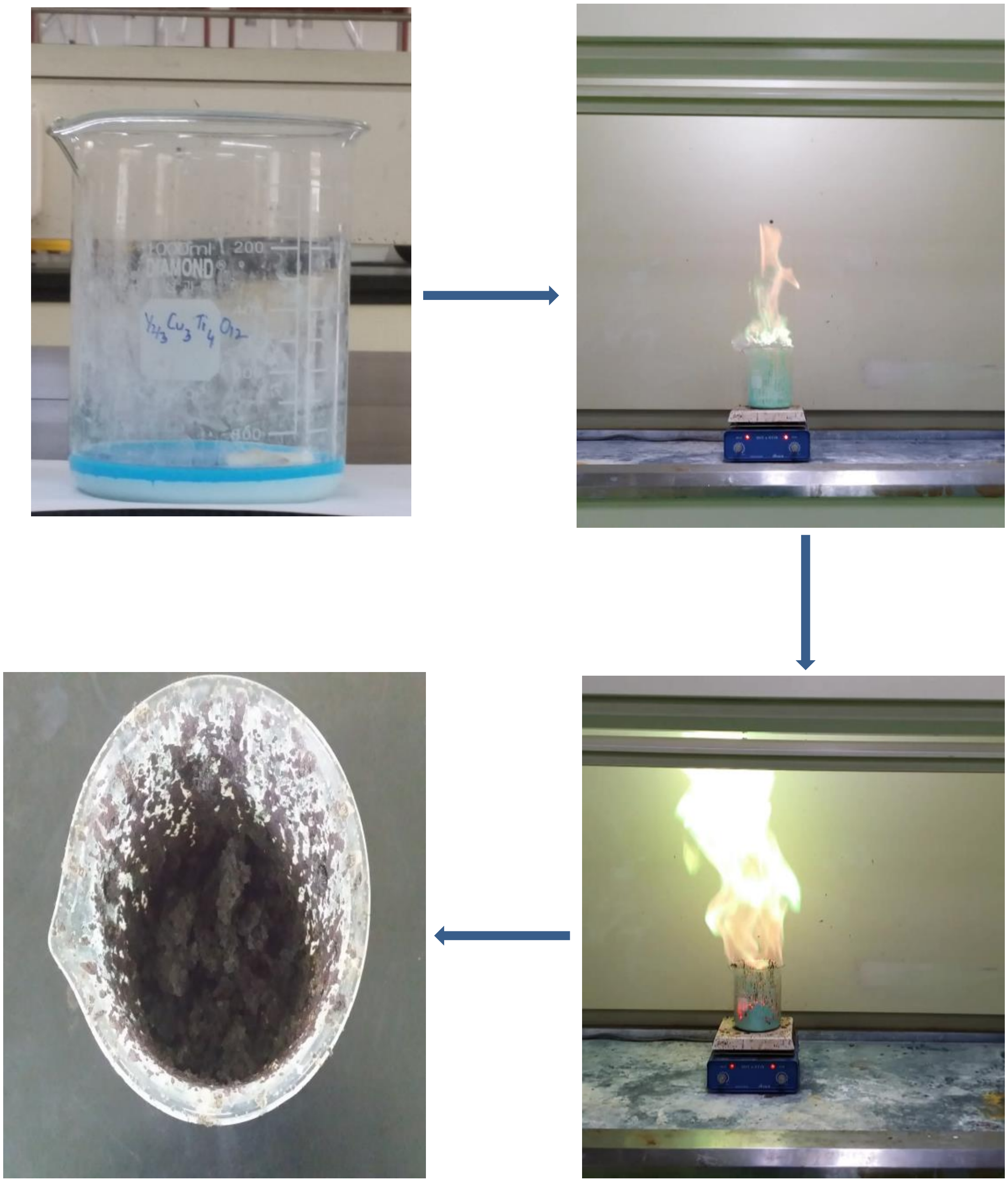

Fig. S3 Photographs taken during the procedure of automatic flame synthesis for $Y_{2/3}Cu_3Ti_4O_{12}$.

Fig. S4

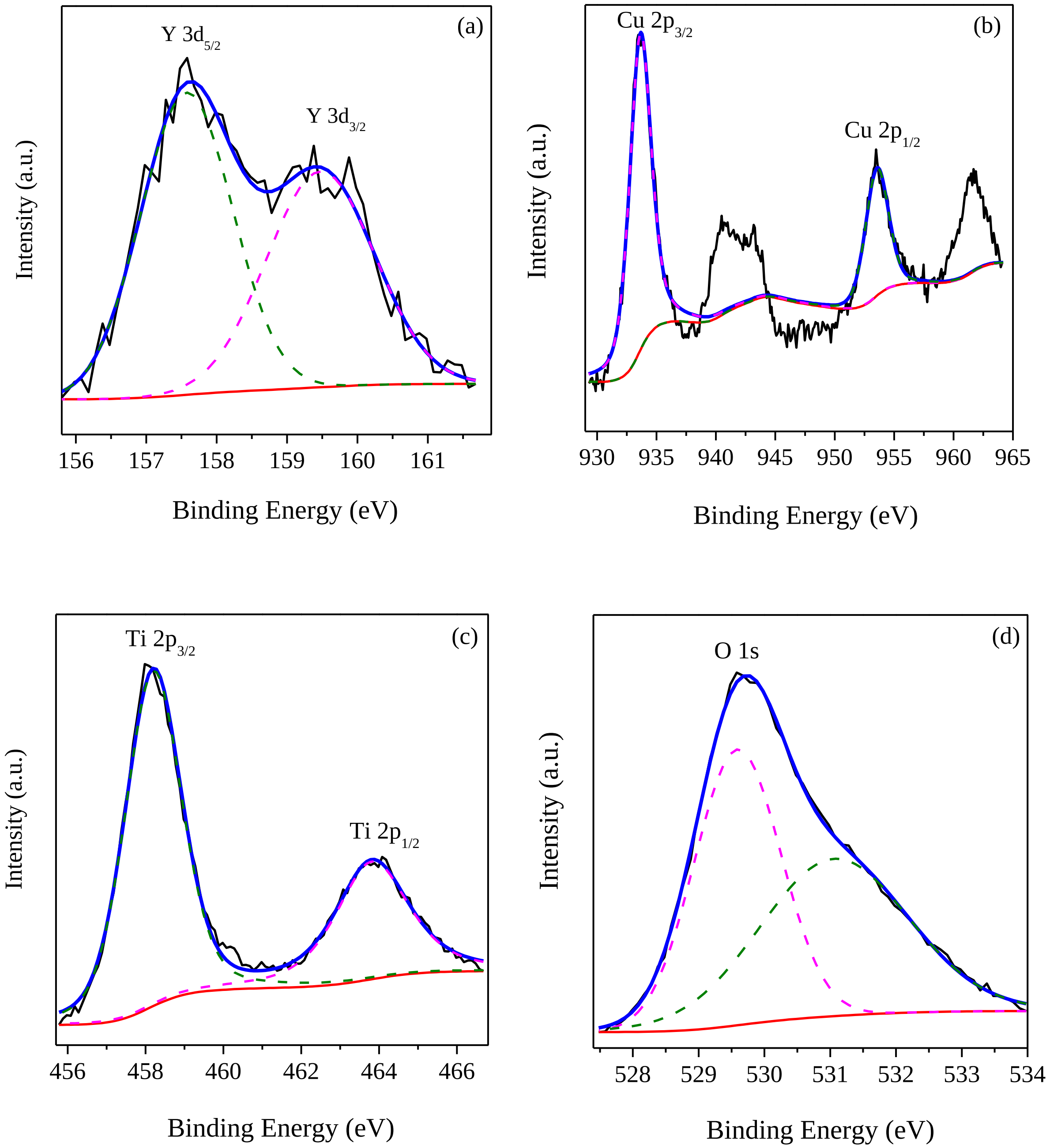

Fig. S4 Fitted XPS spectra of direct sintered $Y_{2/3}Cu_3Ti_4O_{12}$ powder at 1050 °C for 15 hrs: (a) Y3d, (b) Cu 2p, (c) Ti 2p, and (d) O 1s.

Fig. S5

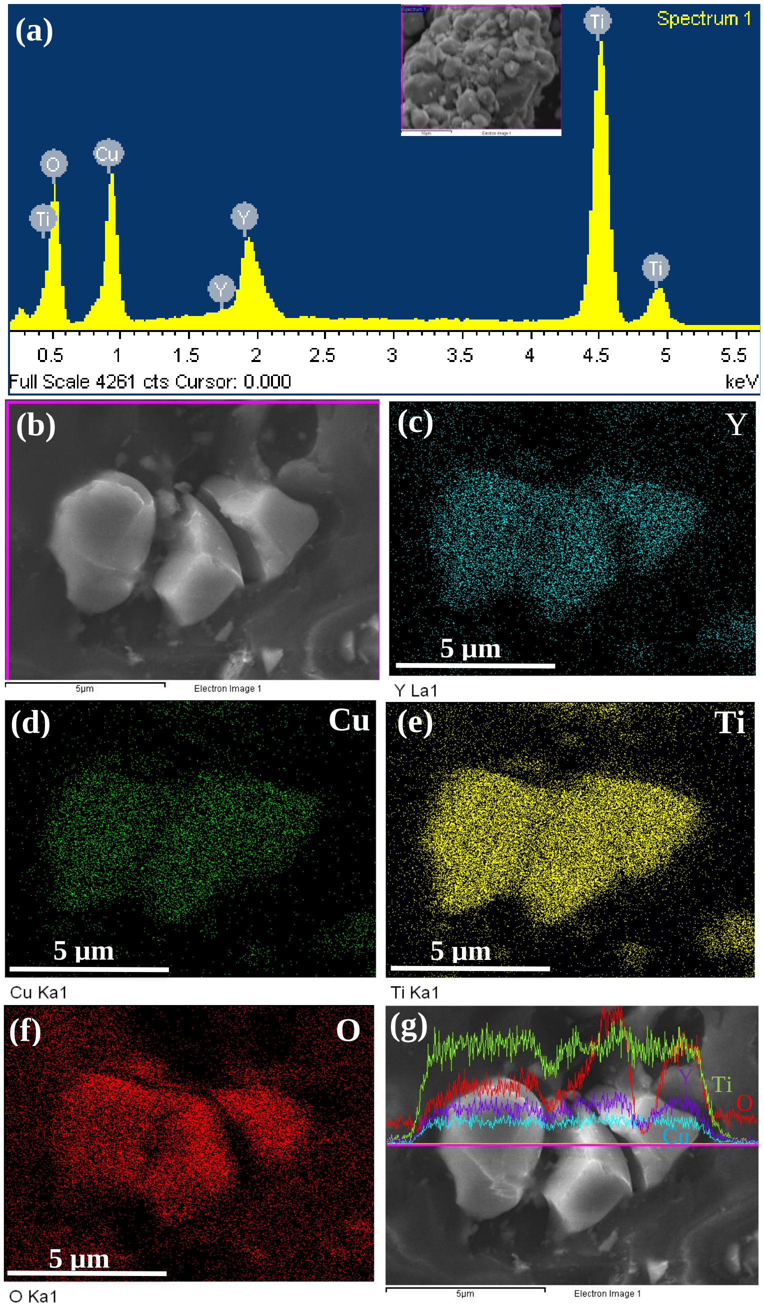

Fig. S5 (a) Energy dispersive spectrum (EDX): Inset shows the selected specific portion. (b) Selected specific SEM image for EDX mapping of (c) Y, (d) Cu, (e) Ti, and (f) O, and for (g) EDX line scan profiles of specific portion of the direct sintered $Y_{2/3}Cu_3Ti_4O_{12}$ powder at 1050 °C for 15 hrs.

Fig. S6

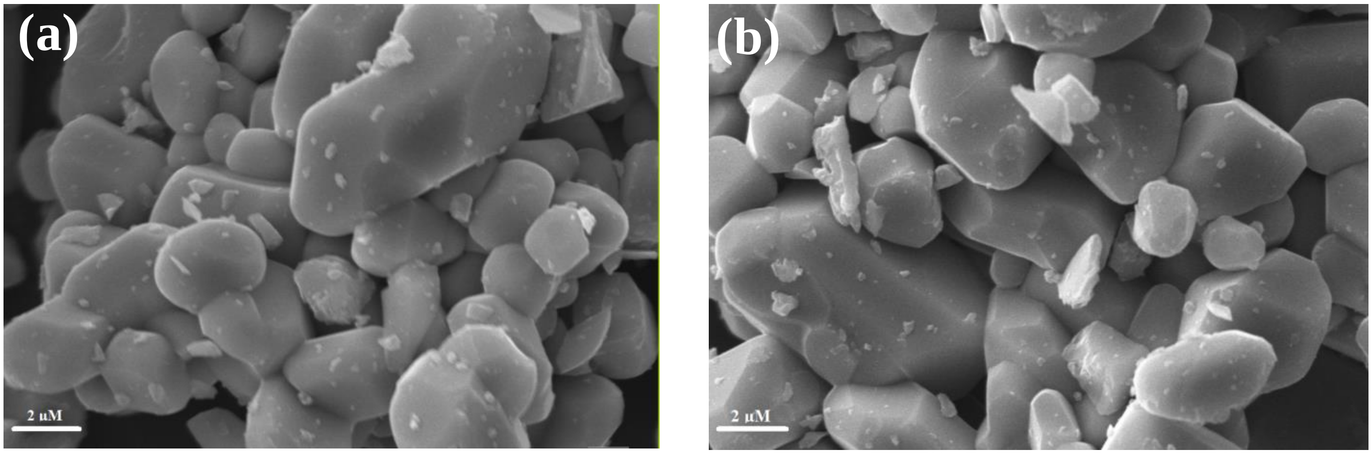

Fig. S6 SEM images of $Y_{2/3}Cu_3Ti_4O_{12}$ (a) before and (b) after 10,000 cycles.

Fig. S7

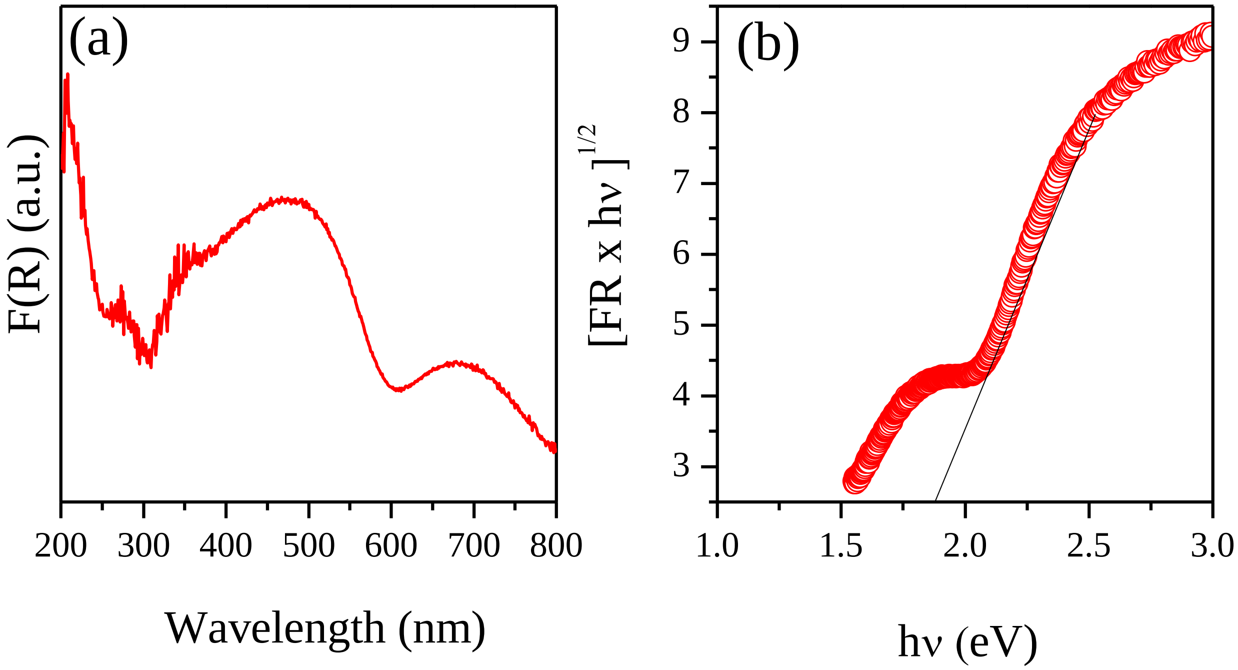

Fig. S7 (a) UV-visible diffuse reflectance spectrum and (b) Kubelka-Munk transformed reflectance spectrum between Kubelka-Munk function $(F(R)h\nu)^{1/2}$ and photon energy (hν) of auto flame synthesized $Y_{2/3}Cu_3Ti_4O_{12}$.
